# Evaluating the performance of a virtual platform ‘T-BOM’ for mentorship in tropical diseases research among early career scientists: Insights from a pilot in Nigeria and other resource-limited settings

**DOI:** 10.1016/j.parepi.2024.e00393

**Published:** 2024-11-20

**Authors:** Hammed Oladeji Mogaji, Akan Itinah, Oyinkansola Suliat Fadiji, Olamide Olaitan Omitola, Tawkalitu Eniola Mogaji, Olajide Murtala Keshinro, Falilat Eniola Mogaji, Mahmud Umar Ali, Moses Aikins, Franklin N. Glozah, Dako-Gyeke Phyllis, Uwem Friday Ekpo

**Affiliations:** a Steering Committee, Top-Bottom Open Mentorship (T-BOM) model, Nigeria; b Federal University Oye-Ekiti, Ekiti State, Nigeria; c Federal University of Agriculture, Abeokuta, Nigeria; d Federal University of Health Sciences, Ila-Orangun, Osun State, Nigeria; e Lagos State University, Ojo, Nigeria; f Aliko Dangote University of Science and Technology, Wudil, Kano State, Nigeria; g School of Public Health, College of Health Sciences, University of Ghana, Accra, Ghana; h TDR Global Africa Node, Ghana

**Keywords:** T-BOM, Research mentorship, Tropical diseases, Scientist, Africa, TDR

## Abstract

**Background:**

Research mentorship plays a crucial role in advancing science. However, there are limited virtual platforms for cultivating mentorship among early career infectious diseases researchers in resource challenged settings. This study reports the findings from the utilization of a recently developed virtual mentorship platform, including its achievements, challenges and needs.

**Methods:**

We developed a web-based application called Top-Bottom Open Mentorship (TBOM) freely accessible at www.tbommodel.com. The platform hosts mentors and allows mentees to send connection requests. In this paper, we present the utilization of this platform, including the opportunities and challenges encountered during the first year of implementation. Utilization data was generated monthly, while opportunities and challenges were captured using a users' perception survey. Data were analyzed in R software and summarized thematically as appropriate.

**Results:**

Between October 2022 and November 2023, the platform registered 81 users, comprising 63 mentees [54.3 % males, 75 % graduate students] from five countries [Nigeria, Cameroon, Brazil, Sudan, and Ghana], and 18 mentors [78 % males] from six countries [Nigeria, USA, Cameroon, Kenya, Brazil, and Tanzania]. Platform engagement increased from 19.4 % (7 users out of 36 who registered) to 51 % (41 users out of 81 who registered) over the year. Also, a total of 16 mentorship cycles were completed, with 9 currently running. Mentees reported having access to job opportunities, enhanced skills in writing, time management, and grant sourcing, and improved research prospects. However, challenges identified include time zone differences, limited number of mentors, mentee's readiness, and associated internet connection issues.

**Interpretation:**

The achievements of T-BOM over a period of one-year are challenged by intrinsic factors from both mentees and mentors, as well as erratic internet services in resource-limited settings. While the platform offers significant opportunities for improving research mentorship, these challenges need to be carefully addressed.

## 1 Background

Over the last two decades, extensive efforts have been made to control and potentially eliminate neglected tropical diseases in low- and middle-income countries (World Health Organization (WHO). Schistosomiasis, 2024). The World Health Organization (WHO) has played a pivotal role by offering essential guidance for establishing national disease control programs (World Health Organization (WHO). Schistosomiasis, 2024; Expanded Special Project for Elimination of Neglected Tropical Diseases (ESPEN). Dashboards, 2024). These programs have been largely successful owing to the number of medicines distributed to endemic communities, as well as burden averted in some previously endemic countries (*Expanded Special Project for Elimination of Neglected Tropical Diseases (ESPEN). Dashboards*, n.d.). An important phase of sustaining and advancing these gains involves the mentorship of early career scientists (Kpokiri et al., 2024). Mentorship has been defined as the professional and non-intimate relationship between an experienced and highly regarded, emphatic person (mentor), who guides a more junior colleague (mentee) in developing specific needs and substantially supporting their personal and professional growth (Okereke, 2000). Most of the experts involved in disease control programs are affiliated with formal institutions such as ministries of health, education or environment, universities and research institutes. Mentorship opportunities for aspiring researchers are therefore available within these settings, but more pronounced in the universities (Noormahomed et al., 2019).

Till date, many institutions in low-middle income countries (LMICs) lack a strong tradition of mentoring (Raghavendran et al., 2017; Chi et al., 2019). Often, relationships are limited to supervision, where a student completes a specific task within a set timeframe as part of degree requirements, unlike mentorship, which typically has no time limits, and is not typically assessed (Olayide et al., 2021). Additionally, the power dynamics in the supervision model are heavily skewed towards the mentors, who primarily serve as instructors and less frequently engage in passive mentoring, while students face most of the demands and expectations (Keller and Lindwall, 2020). Furthermore, access to research mentorship at universities is often hindered by factors such as fear, tuition fees, entrance examinations, distance, especially for marginalized students in LMICs (Olayide et al., 2021; Wagner et al., 2022). In settings where access to mentorship exists, effective mentoring is often limited by fear among students, uncertainty about how to approach and start a mentorship, and conflicts that arise when the same person acts as both a supervisor and a mentor (Ssemata et al., 2017).

Evidently, the need to explore virtual alternatives that address the limitations encountered during traditional supervision or mentorship engagements in the universities have emerged. In 2021, an open call for virtual research mentorship idea contest was organized by TDR Global, Social Entrepreneurship to Spur Health (SESH), and the Armauer Hansen Research Institute (AHRI) (TDR Global Research Mentorship Challenge Contest, 2021). The contest was implemented in stages, first at the regional level in Africa, Asia and South America, and winners at the regional stage, participated in the global contest coordinated by SESH. Our team in Nigeria hypothesized that the use of dynamic virtual platforms can better facilitate mentor-mentee connections beyond traditional settings in the universities, while achieving comparable outcomes. We then developed a web-based mentorship platform, called Top-Bottom Open Mentorship (T-BOM) model, accessible at www.tbommodel.com, and participated in both contests, first with a presentation on our framework, and at the second stage with a proof of concept. We emerged as winners in both contests and received a seed grant to pilot the model from TDR Global African Node. This study therefore presents findings from the utilization of the T-BOM platform, including the opportunities and challenges encountered during the pilot implementation phase.

## 2 Methods

### 2.1 About the T-BOM platform

**Fig. 1:**
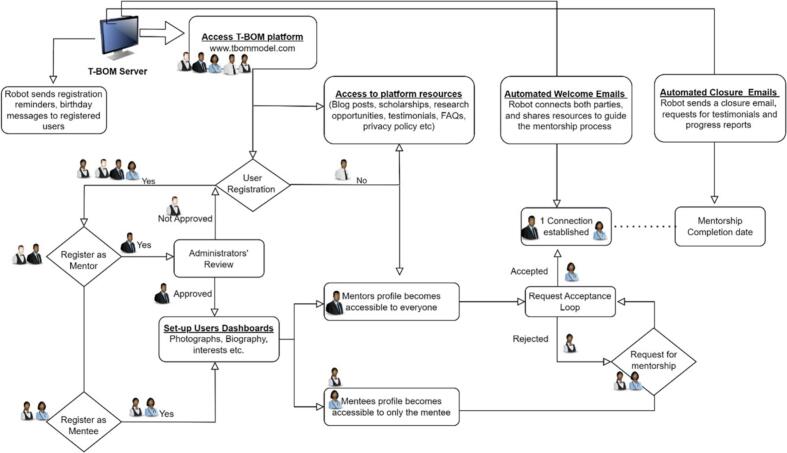
Algorithm that governs the functionality of T-BOM.

T-BOM is a web-based platform designed to facilitate mentor-mentee pairing and to promote mentorship interactions (Fig. 1). The application is easily accessible through a web browser at www.tbommodel.com. The platform utilizes tailored algorithms to perform four primary functions; (i) promote mentor's visibility to mentees, (2) enhance access to mentors with a mentee-driven interaction model, (3) provide flexible interaction platforms and resources to aid mentorship, and (4) introduce an innovative mentorship index for evaluating mentors' performance. The platform has a user-friendly landing page, which features a registration section for new users, and a search bar for locating mentors in specific subject areas. The landing page also serves as a repository of valuable resources such as platform guidelines, codes of conduct, privacy policy, frequently asked questions (FAQs), scholarship, and other research opportunities to support users.

Fig. 1 illustrates the algorithm that governs the platform's functionality. Briefly, users must register on the platform using their email accounts. Upon registration, unique dashboards are automatically created for each user. While mentee registration is straightforward, mentor registration requires verification and prior approval to ensure they are suitably qualified. Mentees are then encouraged to browse through the catalog of mentors and select those whose profiles align with their interests. These profiles include information about the mentor's biography, areas of interest, availability, and preferred platform for mentorship. Behind the scenes, the platform facilitates the exchange of mentorship requests between prospective mentors and mentees. These requests need to be approved by the mentor before a mentorship cycle can be initiated.

Throughout the mentorship process, registered users receive periodic prompt emails, which include links to supplementary resources such as a progress-tracking sheet. To engage unpaired registered users, reminder emails are also sent periodically. Upon completion of the mentorship, the system sends automated closure emails to users, requesting the mentee to rate the mentor's performance and complete a testimonial and progress report sheet.

### 2.2 Enrollment of platform users

The web-based platform became functional and accessible in October 2022 and currently boasts a user base of 81 registered individuals, comprising both mentors (*n* = 18) and mentees (*n* = 63) as of November 2023. Users were recruited through a combination of physical and virtual methods. An open pitch was presented at a widely advertised national conference in Nigeria, while dedicated pages established for the project on online platforms, such as Twitter, Facebook, and LinkedIn, were used to recruit users from other countries.

### 2.3 Evaluation of the T-BOM platform

An evaluation study was designed to assess the functionality of the platform after a six-month pilot implementation phase. This assessment was cross-sectional in approach and included a perception survey among registered users regarding the challenges and opportunities encountered during the period of engagement. Two distinct approaches were employed to comprehensively capture these insights. The initial approach involved an in-depth desk review of monthly reports. This provided an understanding of the progress and dynamics of the process. The second approach encompassed the administration of carefully designed electronic questionnaires (Accessible at https://forms.gle/b6vuEozXViEQmJZg9) to registered users. The questionnaires were tested by two external experts prior to deployment. Invitations to participate were extended to all registered users on the platform irrespective of their level of activity. This inclusivity is driven by the intention to identify barriers to usage among inactive users. To ensure widespread engagement, follow-up emails were sent intermittently over a period of 30 days. The electronic packet shared with the participants included a concise introductory note outlining the objective, a clear consent statement, and the main questionnaire. All questionnaires were designed and self-administered in English language.

### 2.4 Study indicators

We gathered demographic details, including sex, category of registration (mentor/mentee), and country of residence. Additionally, we collected data on (i) how individuals were introduced to T-BOM, (ii) the ratings of their experiences, and (iii) the probability of them suggesting the platform to others. We also inquired if mentors or mentees had ever received requests from mentees or had sent requests to mentors within the last six months, respectively. Among mentors who received requests, we delved further into understanding if they accepted such requests (yes/no). For those who accepted, we posed questions regarding the status of their mentorships (ongoing/completed), if there were any modifications to their mentorship plans (yes/no), and the communication medium they employed (Zoom, WhatsApp, email, etc.). We further explored via open-ended questions the facilitators, challenges, and accomplishments during the mentorship process. On the other hand, mentors who never accepted such requests were prompted to provide the primary reasons for their decisions (e.g., forgetfulness, busy schedules, divergent interests, and platform-related issues). A comparable approach was also employed to collect information from mentees.

### 2.5 Ethical considerations

The Institutional Review Boards (IRBs) often consider these surveys as part of monitoring process; therefore, an IRB does not require that it provide ethical oversight of the survey. However, approvals were sought from the Institutional Review Board at the Federal University Oye-Ekiti (FUOYEFSC 201122 –.

REC2024/019), and additional written consents were obtained from registered users (electronically) prior to completion of the questionnaires. Participation in this survey was made voluntary. Data were collected anonymously to protect participants. Participants were also informed that data gathered from the survey will be used to evaluate the platform's performance and shared widely via conferences and publications.

### 2.6 Data management and analysis

Data collected were imported into R software version 4.3.2 for analysis. Descriptive statistics including frequencies and percentages, were used to summarize categorical data. Qualitative data from open-ended responses were organized in Microsoft Excel (v. 2007), coded, and analyzed thematically. The outcomes of the analysis were presented using graphs to visualize the results and narrative text to provide explanations and insights.

## 3 Results

### 3.1 Website activity and associated metrics

**Fig. 2:**
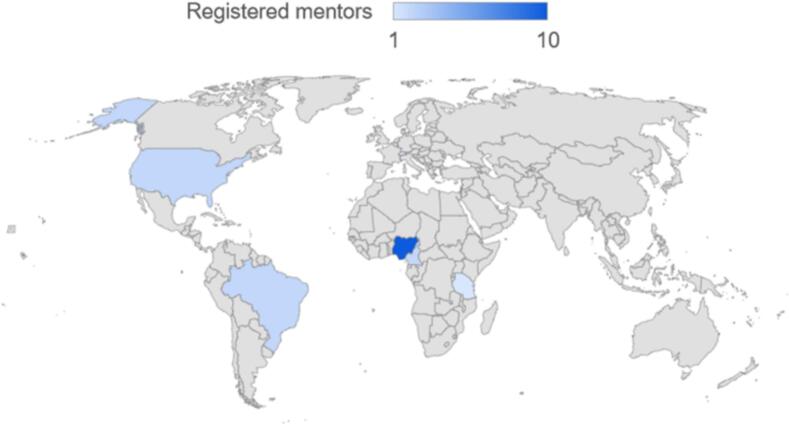
Distribution of registered mentors by countries.

**Fig. 3:**
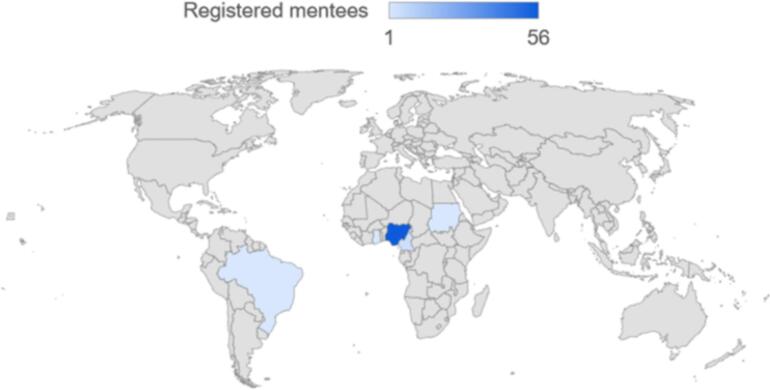
Distribution of registered mentees by countries.

**Fig. 4:**
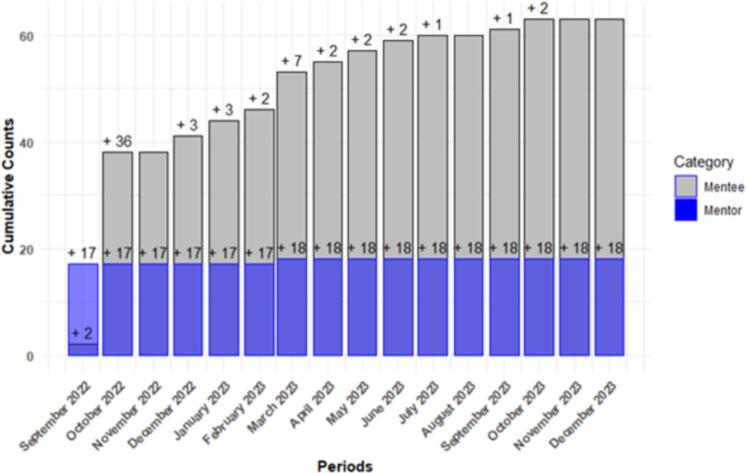
Enrollment of mentors and mentees over a 12-month period.

Between September 2022 and November 2023, a total of 81 users were registered on the platform. Out of these, 63 were mentees from five different countries, while 18 were mentors from six countries (Table 1, Fig. 2, Fig. 3). By gender, 77.8 % of the mentors and 54.3 % of the mentees were males, and by educational qualifications, majority of the mentors were Ph.D. holders (77.8 %), while for the mentees, most were M.Sc. and B.Sc. degree holder with 42.9 % and 32.9 % respectively. There is a remarkable 125 % increase in the number of registered users within a 12-month period from the starting month(Fig. 4).

**Table 1:** Summary of registered users on the TBOM platform.

	Mentors		Mentees		Total	
	N	%	N	%	N	%
Gender						
Female	4	22.2	33	52.4	37	45.7
Male	14	77.8	30	47.6	44	54.3
Total	18	100	63	100	81	100

Country						
Brazil	2	11.1	1	1.6	3	3.7
Cameroon	2	11.1	4	6.3	6	7.4
Switzerland	1	5.6	0	0.0	1	1.2
Nigeria	10	55.6	56	88.9	66	81.5
Tanzania	1	5.6	0	0.0	1	1.2
USA	2	11.1	0	0.0	2	2.5
Sudan	0	0.0	1	1.6	1	1.2
Ghana	0	0	1	1.6	1	1.2
Total	18	100	63	100	81	100

Qualifications						
Ph.D. (Professor)	3	16.7	0	0.0	3	3.7
Ph.D.	14	77.8	13	20.6	27	33.3
M.Sc.	1	5.6	27	42.9	28	34.6
B.Sc.	0	0.0	22	34.9	22	27.2
Others	0	0.0	1	1.6	1	1.2
Total	18	100	63	100	81	100

**Fig. 5:**
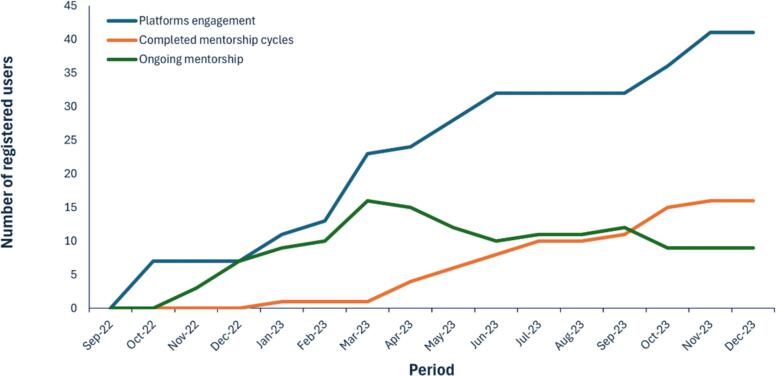
Overview of mentorship cycles initiated over a 12-month period.

Most users who registered as mentees were from Nigeria (*n* = 56), followed by Cameroon (*n* = 4), Brazil (*n* = 1), Sudan (n = 1), and Ghana (n = 1) (Fig. 2, Table 1). Similarly, the platform attracted 18 mentors from Nigeria (*n* = 10), the United States (*n* = 2), Cameroon (n = 2), Brazil (n = 2), Switzerland (n = 1), and Tanzania (n = 1) (Fig. 3, Table 1). Engagement on the platform experienced substantial growth, increasing from 7/36 (19.4 %) in the first month to 41/81 (51 %) over the 12-month period (Fig. 4). Additionally, there were a total of 16 completed mentorship cycles within the 12 months period, with 9 active/ongoing cycles (Fig. 5).

### 3.2 Profile of participants responding to evaluation survey

Of the 76 registered users, 33 (33.4 %) responded to the online survey. Among these respondents, 12 of 18 (67 %) identified themselves as mentors, while 21 of 57 (37 %) identified themselves as mentees. Most mentees were female (*n* = 12), while mentors were male (*n* = 8). By countries, mentors were from Nigeria (n = 6), Brazil (n = 2), Cameroon (*n* = 1), the United States (*n* = 2), and Tanzania (n = 1). However, mentees were from Nigeria (*n* = 20) and Ghana (n = 1).

### 3.3 Functionality of the web-based application

Participants rated their experience on the platform, with an average rating of 6.24 out of 10. Additionally, when asked about the likelihood of recommending the site to others, participants indicated an average rating of 7.48 out of 10. The primary methods through which participants learned about the application were as follows: word of mouth, which accounted for 36.36 % (12 mentions); email requests, 12.21 % (seven mentions); organized pitch, 15.15 % (five mentions); WhatsApp, 9.09 % (three mentions); and Twitter, 6.06 % (two mentions). Furthermore, most participants, totaling 84.38 % (27 individuals), reported not encountering any difficulties with the website, indicating a smooth user experience. (Table 2).

**Table 2:** Functionality of the web-based application.

*N* = 33	Frequency Or Mean	%
Users' experience on a scale of 10	6.24	–
Likelihood of recommending the website to others	7.48	–
How did you hear about the website?		
Word of mouth	12	36.36
Email Request	7	21.21
Pitch	5	15.15
WhatsApp	3	9.09
Twitter (X)	2	6.06
Others	4	12.12

Had trouble with the website		
None	27	84.38
Message in spam folder	1	3.13
Did not use it	1	3.13
Yes, with the search bar	1	3.13
No response	2	6.25

### 3.4 The behavioral pattern of mentors and mentees

**Fig. 6:**
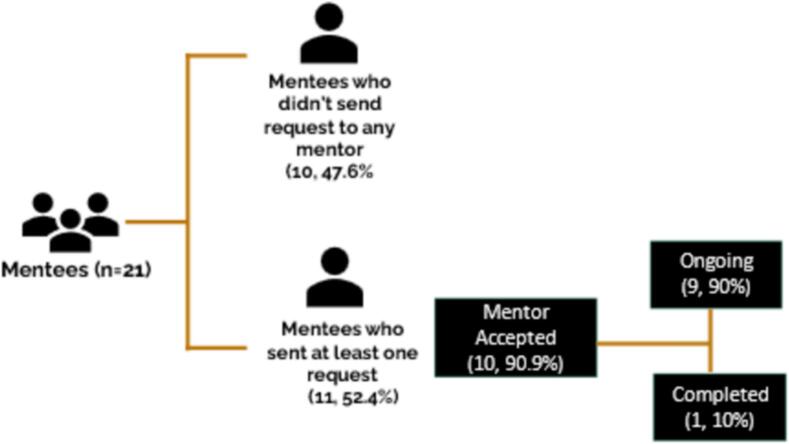
Behavioral pattern of mentees during the pilot phase.

**Fig. 7:**
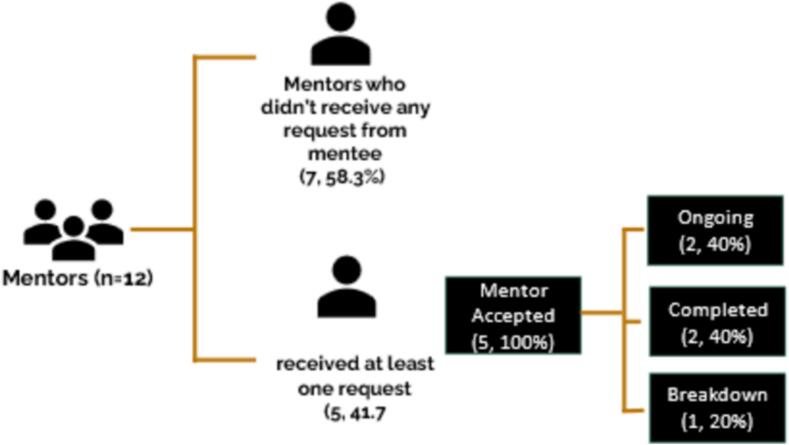
Behavioral pattern of mentors during the pilot phase.

Of the 21 mentees who responded, 47.6 % (10 mentees) did not utilize the platform. Among this group, 60 % mentioned forgetting or facing difficulties in navigating the website, while 40 % attributed this to business or other reasons. Among those who engaged with the platform, the majority (90.9 %) requested a mentor. Notably, 10 % completed the mentorship, while 90 % had an ongoing mentorship (Fig. 6). Similarly, among the responding mentors, 58.3 % (seven mentors) claimed that they did not receive requests from mentees. However, 43 % of them (three mentors) received such emails. There are potential explanations, such as emails landing in the SPAM box, mentors forgetting, being busy, or intentionally ignoring the requests (Fig. 7). Nevertheless, all mentors who acknowledged receiving at least one request from a mentee accepted the request (100 %). Among them, 40 % completed their mentorship, 40 % had ongoing mentorships, and 20 % experienced a breakdown in communication (Fig. 7).

### 3.5 Enablers promoting users' engagement

The analysis of open-ended responses from mentors and mentees revealed factors that contributed to the success of their mentorship cycle.

Mentor Availability and Responsiveness.

Many respondents emphasized the importance of the mentor being accessible, available, passionate and responsive. This includes prompt communication and the mentor's commitment to helping the mentee.

“My number one enabler is the willingness and availability of one of my mentors [name withheld] to attend and respond to my help. He has shown and demonstrated that leadership style with his prompt response as always.” – **Mentee from Nigeria.**

“Being available and responsive to constraints beyond human control” – **Mentor from Switzerland.**

### 3.6 Mentor expertise and teaching style

The mentor's knowledge, experience, and how they convey information (teaching style) play a vital role in effective mentorship.

“[Name withheld] expertise, passion, teaching style, structured and regular meetings, practical assignments with the ability to make corrections to every given assignment as well as his patience and willingness to answer questions and provide me with the necessary resources to learn and grow, and teaching of valuable skills….”- **Mentee from Nigeria.**

Use of Technology (WhatsApp).

The use of WhatsApp was highlighted as a key facilitator in the mentorship process due to its convenience, accessibility, and low cost.

“The WhatsApp platform was more time-friendly and cheaper”- **Mentor from Brazil.**

### 3.7 Continuous support and guidance

Persistent communication and mentorship guidance, such as career advice (e.g., pursuing further education), were also essential for maintaining engagement and development.

“Persistent communication, Advice from my mentor on furthering my education to PhD level.” – **Mentee from Nigeria.**

Challenges limiting users' engagement.

The analysis of open-ended responses from mentors and mentees revealed factors that challenged the mentoring process.

Time Zone Differences and Scheduling Conflicts.

Mentees and mentors struggled with coordinating meetings and communication due to time zone differences and conflicting schedules.

“One of the challenges is timing, especially for mentors in different locations of the world apart from where their mentees reside”- **Mentee from Nigeria.**

“Time constraints, Unavailability of my mentor, Communication barriers, Busy schedule of my mentor”- **Mentee from Nigeria.**

“Busy schedules” – **Mentor from Switzerland.**

### 3.8 Limited mentor availability

Mentors' busy schedules and high work demands often restricted their availability, which negatively impacted the mentorship experience.

“Technical issues such as …. the busy Schedules of my mentor in a few times were the major challenges” – **Mentee from Nigeria.**

“Availability due to high work demands for myself, and also the availability/readiness of the mentees. Some of the mentees whom I had accepted their request never reached out” – **Mentor from Brazil.**

### 3.9 Poor internet connectivity and infrastructure

Both mentees and mentors reported that poor internet connectivity and issues such as electricity outages created significant obstacles.

“The internet network and poor electricity supply”. – **Mentor from Nigeria.**

“High internet time” – **Mentor from Nigeria.**

“Technical issues such as poor internet connectivity…… were the major challenges” – **Mentee from Nigeria.**

### 3.10 Mentee readiness and engagement

One mentor highlighted that mentees were not always prepared or proactive in engaging in the mentorship process, which posed a challenge to effective mentorship.

“…… availability/readiness of the mentees. Some of the mentees whom I had accepted their request never reached out” – **Mentor from Brazil.**

### 3.11 Achievements of participants

Several mentees underscored their accomplishments during the pilot phase of the project. Notable achievements include the following.

### 3.12 Skill development and knowledge enhancement

Mentees highlighted significant improvements in their skills, particularly in areas such as research management, critical thinking, writing, and data analysis. These skill developments contributed to their professional growth.

“Deeper understanding of public health/parasitology, increased critical thinking skills, improved time management skills, improved data analysis skills, and professional development in abstract writing” – **Mentee from Nigeria.**

“Trained the mentee on how to write quality abstract with limited word count, how to use SPSS for basic analysis and how to effectively manage time”- **Mentor from Nigeria.**

### 3.13 Successful grant proposal writing and submissions

Several participants, both mentors and mentees, noted the successful completion and submission of grant proposals as a major achievement, demonstrating the impact of mentorship on research advancement.

“writing of grant proposal” – **Mentee from Ghana.**

“Submission of Grants for 3 of my mentees; Inclusion of my mentee in an ongoing fieldwork”- **Mentor from Brazil.**

“Through this mentorship, I was able to submit grant proposals to RSTMH and TDR…..” – **Mentee from Nigeria.**

### 3.14 Participation in paid research opportunities

Mentees benefited from active participation in paid research opportunities, particularly involving fieldwork, which helped them gain practical experience and broadened their research exposure.

“……Inclusion of my mentee in an ongoing fieldwork”- **Mentor from Brazil.**

### 3.15 Mentorship in research management and coordination

Some mentees highlighted their achievements in taking on research assistant and coordinator roles, learning how to manage research programs, and being introduced to specific research fields such as implementation research and neglected tropical diseases.

“My key achievement is the opportunity to be mentored by [name withheld] in handling a research program as a research assistant and research coordinator. I was introduced by him to the field of Implementation research and neglected tropical disease….”.- **Mentee from Nigeria.**

### 3.16 Establishing professional interactions

Mentors and mentees pointed out that the mentorship facilitated the establishment of personal and professional interactions, which fostered collaboration and personal development.

“Established personal interactions and reviewed a grant proposal.” – **Mentor from Switzerland.**

### 3.17 Recommendations from users

Recommendations for enhancing mentorship through the platform were primarily centered on the following.

### 3.18 Increased publicity and awareness

Many users recommended greater visibility for the platform through targeted publicity efforts, particularly on academic websites and social media. This would attract both mentees and mentors and stimulate demand for mentorship.

“My major recommendation is to boost up its awareness for like-minded people because i feel most people are unaware of this great platform of which they will jump on instantly when they get to know about it. “– **Mentee from Nigeria.**

“More visibility of the platform to both mentees and mentors, More convincing strategies to promote demand/readiness among the mentees” - **Mentor from Brazil.**

“More publicities on academic websites” - **Mentor from Nigeria.**

“Social media awareness, Academic writing on the platform and social engagement”.– **Mentee from Nigeria.**

“Increase awareness to get people to use and be aware of the platform” ***– Mentor from United States of America.***

### 3.19 Strategies to increase mentee engagement

Users emphasized the need to develop strategies that would encourage more active participation from mentees, including readiness and demand for mentorship. Suggestions included structured reminders and follow-up emails to track progress.

“………More convincing strategies to promote demand/readiness among the mentees” - **Mentor from Brazil.**

“Reminder and a follow-up email prompt will be good if used to monitor the progress of the interaction between Mentor and mentee.” - **Mentor from Nigeria.**

### 3.20 Improvement in platform features and responsiveness

Several users recommended enhancing the functionality and user experience of the platform, such as making the website more mobile-responsive and adding features like a mentee rating system to provide mentors with feedback.

“Make website more mobile responsive” – **Mentee from Nigeria.**

“Introduce a rating system of mentees for the mentors…...”- **Mentee from Nigeria.**

“…..I think the website itself needs to be more engaging for it's users”. *……”- **Mentee from Nigeria.***

“Mentors and mentees should be provided with resources to help them make the most of their relationship. This could include training on how to mentor or be mentored, as well as access to support materials.” – **Mentee from Nigeria.**

### 3.21 Expanding resources and mentor availability

Users suggested that the platform should offer more resources and materials related to mentorship, while also increasing the number of available mentors and expanding the range of research topics.

“…………….Increase the number of mentors, provide more resources and materials, increase the range of available research interests or topics …...”- **Mentee from Nigeria.**

“Seek to provide information on training and grants opportunities”**- Mentor from Switzerland.**

Getting more mentors, acquiring more mentees, being able to cut across other areas of science world” **– Menteee from Nigeria.**

“Update List of mentors and mentees” - **Menteee from Cameroon.**

### 3.22 Use of periodic online meetings for engagement

Periodic online meetings were proposed to introduce and communicate the platform's objectives to both mentors and mentees, as well as to foster engagement through advocacy and branding initiatives.

“…… Online meetings to introduce objectives to mentees and mentors; Advocacy among the targeted group; Brand souvenir to increase awareness”– **Mentor from Nigeria.**

“Increase publicity, Periodic online presentations on the T-BOM platform.” **– Mentee from Nigeria.**

## 4 Discussion

Over the past four decades, the Special Program for Research and Training in Tropical Disease (TDR), jointly supported by 10.13039/100006641UNICEF, UNDP, 10.13039/100004421the World Bank, and WHO, has pursued dual objectives – first is to enhance research capabilities in regions where infectious diseases disproportionately affect disadvantaged populations, and to prioritize and finance research endeavors aimed at combating these diseases (Vahedi et al., 2015; Reeder et al., 2024). Their efforts have significantly contributed to enhancing the health and well-being of individuals afflicted by infectious diseases in impoverished regions across Africa, Asia, and Latin America (Reeder et al., 2024; Kaba et al., 2023; Joshi et al., 2023; Reeder, 2014). Recently, there has been a concerted focus on fostering research mentorship as a pivotal strategy for cultivating a larger cohort of scientists in low- and middle-income countries (LMICs), thereby equipping them with the requisite skills to sustain ongoing endeavors aimed at improving the health and welfare of vulnerable populations (Reeder, 2014; World Health Organization, 2024). Noteworthy investments in the past decade have included a particular emphasis on implementation research (World Health Organization, 2024). Since 2013, the program has consistently organized workshops, launched massive open online courses (MOOCs), established regional training centers, and funded postgraduate training programs across LMICs. While these initiatives are crucial, the program has also encouraged the generation of innovative research mentorship concepts through open competitions (TDR Global Research Mentorship Challenge Contest, 2021), and has developed the HERMES guide to outline strategies for institutionalizing research mentorships (World Health Organization, 2022).

While research mentorship has been acknowledged as a lifelong process with the potential to advance the global health agenda (Reeder, 2014; World Health Organization, 2024; World Health Organization, 2022; Lescano et al., 2019), significant barriers continue to impede its implementation in low- and middle-income countries (LMICs) (Kaba et al., 2023). One of the foremost erroneous assumptions in LMICs is that “supervising” is synonymous with “mentorship.” (Olayide et al., 2021; Lescano et al., 2019) Consequently, most relationships between supposed mentors and mentees have been confined to “supervisorship.” Furthermore, the scarcity of mentors with extensive mentoring training, a cultural imbalance favoring hierarchical models over the horizontal practices seen in high-income countries, and a lack of awareness, institutional support, and resource allocation have been reported (Kaba et al., 2023; Lescano et al., 2019; Bain et al., 2023). These challenges are exacerbated in marginalized settings even within LMICs, where there are significant access barriers (language, distance and cost) to institutions offering mentorship (Bain et al., 2023)However, the evolution of digital technologies has created new opportunities for mentorship (Bain et al., 2023; Cole et al., 2016). There are accounts of hybrid mentorship models, where mentors utilize virtual messaging platforms to establish connections with their mentees (Bain et al., 2023). While this method helps overcome obstacles to engagement, it doesn't directly tackle the challenges associated with seeking mentors, initiating mentorship and appropriate matching between entirely new parties (mentors and mentees) (Olayide et al., 2021; Wagner et al., 2022; Ssemata et al., 2017; Bain et al., 2023). The lingering questions remain: how and where does a potential mentee find a mentor?; Who is responsible for initiating the mentorship process?; and, how should one kick-start the process?.

Hence, in this project, we developed, and piloted a complementary virtual platform designed to promote research mentorship in LMICs. Our initiative is based on the premise that a virtual platform can significantly eliminate barriers to access, especially for marginalized populations who may lack appropriate resources to enroll in a university of choice, or engage with a mentor of choice (Olayide et al., 2021; Wagner et al., 2022; Ssemata et al., 2017). We further hypothesized that a mentee-driven virtual mentorship platform with a mentor rating system would demystify the hierarchical model of engagement and promote horizontal exchanges between mentors and mentees (Lescano et al., 2019). We hereby discuss the findings from the implementation of the TBOM model under the dimensions of functionality, coverage, achievements or relative benefits, and challenges encountered.

Foremost, the functionality of a virtual mentorship platform is pivotal for its adoption and sustainability. Our assessment, based on user ratings and potential referrals, yielded favorable outcomes. On average, the platform garnered a rating and referral score of 62 % and 75 %, respectively. While there are no comparable platforms or benchmarks for reference, ratings surpassing 50 % suggest that the platform's algorithms, aesthetics, and workflows meet satisfactory standards. However, virtual platforms, unlike traditional in-person meetings, are dynamic and rely on software programs and written codes that require ongoing monitoring and updates, especially when modifications are made by source providers. The hosting, continuous oversight and management of virtual mentorship platforms therefore incur operational expenses, in contrast to regular virtual communication platforms. Nevertheless, these costs may be more economical compared to in-person meetings, which are localized, and often entail expenses such as venue rental, maintenance, travel, and refreshments.

Secondly, one important benefit of virtual platforms is their capability to reach very new populations, which might have been missed by traditional in-person programs, owing to previously highlighted access barriers (Bain et al., 2023; Cole et al., 2016). We explored physical and virtual advocacy streams because of the limited budget for advocacy. Hence our most intense publicity was done through Twitter (X), a very common social media messaging platform. However, our findings revealed most of those enrolled into the platform heard through word of mouth from friends and colleagues. We had a total of 81 users (mentors and mentees) across 6 countries from Africa, South America and North America continents. The enrollment rate notably surged by nearly 60 %, and 10 mentorship cycles were completed. This success—evident in coverage, mentor matching, and mentorship completion—can be partly attributed to the platform's in-built algorithms and robot. Upon registration, the robot creates users' dashboards, curates and openly displays mentor's profile to prospective mentees, giving them the freedom to choose their mentors. Additionally, the built-in robot sends monthly reminders and resource tools to support the mentorship process, further assisting in monitoring progress and tracking outcomes. These features are largely absent or less automated in traditional in-person mentorship programs in most LMICs (Bain et al., 2023; Cole et al., 2016).

Furthermore, we aimed to investigate whether the benefits of virtual mentorship would align with those of traditional in-person mentorship. Our findings revealed that mentees gained new career connections, improved skills, job opportunities, and successfully wrote and applied for research grants. These accomplishments are comparable to those reported from in-person mentorship programs (Cole et al., 2016; Speer et al., 2021). Findings from our perception survey on key enablers suggest that the success of a virtual mentorship platform largely depends on the number, availability, and skills of the mentors. Even with a robust platform and eager mentees, the mentor remains a crucial element. While virtual mentorship platforms can offer social, academic, and career support and foster the development of transferable and technical skills like those gained through in-person mentoring, they also provide increased flexibility in meeting times and mediums, the ability to record interactions, and a more comfortable environment for mentee communication (Ensher et al., 2003; Cantrell et al., 2010; Owen, 2015). However, a major challenge has been the access to compatible technologies for both parties and the associated costs (Speer et al., 2021). In our case, the use of flexible communication platforms such as WhatsApp was beneficial in connecting mentors and mentees. WhatsApp use has become increasingly common in healthcare due to its ability to connect individuals globally with their mobile phone numbers, even when they cross international borders (Manji et al., 2021; Morris et al., 2021; Nikolic et al., 2018). However, concerns about privacy remain, and there have been suggestions to optimize the platform to include real-time messaging and video meeting features.Despite our achievements, we encountered several setbacks that limited the program's effectiveness. Foremost, only 50 % of the registered mentors on the platform were active (i.e., either receiving or accepting requests), while mentees' activity was even lower at 25 % (i.e., those that sent requests). Since the model is currently mentee-driven, the engagement of mentors is directly influenced by the activeness of mentees. Studies have highlighted the importance of mentee persistence for effective mentorship, making it crucial to address issues affecting mentee readiness, even with a dynamic platform like ours (McGee and Keller, 2007).

Secondly, challenges such as poor internet connectivity, high internet costs, time zone differences, and the busy schedules of mentors significantly hinder the effectiveness of the T-BOM platform (Bain et al., 2023). While addressing internet-related issues may be beyond the platform's control, these challenges are common in resource-limited settings and reflect broader socio-economic development issues in LMICs. These systemic barriers often exceed the influence of individual researchers or funders. Nevertheless, user feedback has provided several potential solutions, including improving the platform's accessibility and user-friendliness to mitigate some of these challenges. Furthermore, we emphasized that the platform offers tangible benefits, making the investment of time and resources worthwhile. For example, mentees have reported significant achievements, such as successful grant submissions and participation in research projects, which highlight the platform's value. To further enhance mentor participation, we propose introducing a reward system that acknowledges mentors' contributions and helps to offset some of the costs they incur. These practical recommendations aim to bolster the sustainability and effectiveness of the platform, even in the face of these systemic challenges.

Thirdly, the low number of mentees enrolled on the platform reflects limited awareness. Addressing this could benefit from advocacy efforts aimed at increasing demand among potential mentees by showcasing the achievements of previous mentees on the webpages of academic institutions and affiliated societies, such as the American Society of Tropical Medicine and Hygiene (ASTMH), American Society for Microbiologist (ASM), Royal Society for Tropical Medicine and Hygiene (RSTMH), TDR, International Society for Infectious Diseases (ISID), TDR, and others. Institutionalizing the program at academic institutions could help expand the pool of mentors within each region, potentially mitigating time zone issues. Additionally, offering rewards for mentors—such as discounted memberships in scientific societies, grants for conference attendance, or waivers for article processing charges—could support their professional growth and encourage greater participation and referrals. These recommendations require substantial investment, achievable only through partnerships with professional, scientific societies and academic institutions.

On the other hand, it is important to recognize that mentorship is an ongoing and iterative process, and the most important achievement is often the relationship built between mentors and mentees, which, when sustained, can yield long-term benefits beyond the short-term benefits during formal mentorship period. A potential area for future research would be to conduct follow-up studies to investigate the proportion of mentor-mentee relationships that continue after the mentorship cycle and the outcomes they produce.

Nevertheless, one major limitation of this evaluation is the low representation of mentees from countries other than Nigeria. Most survey respondents were from Nigeria, with only one mentee from Ghana. While we highlight that the platform was designed to be accessible globally. The bias towards Nigeria is largely due to the funding constraints, which limited our publicity to virtual methods, and one in-country (physical) event. For future phases, we recommend a more localized and institutionalized approach to advocacy and recruitment. We acknowledge that low representation may limit the generalization of responses, however the majority of issues identified as barriers are common for virtual engagement platforms.

## 5 Conclusion

The implementation and pilot testing of the T-BOM virtual mentorship platform showed promising potential in overcoming access barriers and fostering research mentorship in LMICs. The achievements recorded in 12 months are comparable to those reported in traditional in-person mentorship programs and are dependent on mentee readiness and mentors' availability and skills. Persistent challenges include internet connectivity and time zone differences. Our findings suggest that more detailed advocacy, showcasing the achievements of mentees, increasing pool of mentors, and providing incentives for mentors would support the readiness and retention of both mentees and mentors. The platform promises to be an effective complementary mentoring tool; however, ensuring sustainability and impact will require significant partnership, investment and collaboration with scientific and academic societies.

## Consent for publication

No personally identifying information was included in this research.

## Availability of data and materials

The datasets generated and analyzed during this study have the provided as supplementary files.

## Funding

This study was supported by TDR, the UNICEF/UNDP/10.13039/100004421World Bank/WHO Special Programme for Research and Training in Tropical Diseases through a TDR Global grant.

## CRediT authorship contribution statement

**Mogaji Hammed Oladeji:** Writing – review & editing, Writing – original draft, Resources, Project administration, Methodology, Investigation, Funding acquisition, Formal analysis, Data curation, Conceptualization. **Itinah Akan:** Writing – review & editing, Software, Resources, Data curation. **Fadiji Oyinkansola Suliat:** Writing – review & editing, Supervision, Project administration, Methodology, Investigation. **Omitola Olamide Olaitan:** Writing – review & editing, Supervision, Methodology, Investigation. **Mogaji Tawkalitu Eniola:** Writing – review & editing, Supervision, Project administration, Methodology, Investigation. **Keshinro Olajide Murtala:** Writing – review & editing, Supervision, Methodology, Investigation, Data curation. **Mogaji Falilat Eniola:** Methodology, Project administration, Resources, Writing – review & editing. **Ali Mahmud Umar:** Writing – review & editing, Validation, Supervision, Methodology, Investigation. **Aikins Moses:** Writing – review & editing, Validation, Supervision, Methodology, Investigation, Funding acquisition. **N. Glozah Franklin:** Writing – review & editing, Validation, Supervision, Methodology, Investigation, Funding acquisition. **Phyllis Dako-Gyeke:** Writing – review & editing, Supervision, Methodology, Investigation, Funding acquisition. **Ekpo Uwem Friday:** Writing – review & editing, Validation, Supervision, Project administration, Methodology, Investigation, Formal analysis, Conceptualization.

## Declaration of competing interest

ALL the above-mentioned authors declare there are no conflict of interest applicable to this research study and the work presented.
